# An investigation into the unusual linkage isomerization and nitrite reduction activity of a novel tris(2-pyridyl) copper complex

**DOI:** 10.1098/rsos.170593

**Published:** 2017-08-16

**Authors:** Isolda Roger, Claire Wilson, Hans M. Senn, Stephen Sproules, Mark D. Symes

**Affiliations:** WestCHEM, School of Chemistry, University of Glasgow, University Avenue, Glasgow G12 8QQ, UK

**Keywords:** nitrite reduction, linkage isomerism, copper nitrite reductase mimic, electrocatalysis, nitrite binding mode

## Abstract

The copper-containing nitrite reductases (CuNIRs) are a class of enzymes that mediate the reduction of nitrite to nitric oxide in biological systems. Metal–ligand complexes that reproduce the salient features of the active site of CuNIRs are therefore of fundamental interest, both for elucidating the possible mode of action of the enzymes and for developing biomimetic catalysts for nitrite reduction. Herein, we describe the synthesis and characterization of a new tris(2-pyridyl) copper complex ([Cu**1**(NO_2_)_2_]) that binds two molecules of nitrite, and displays all three of the common binding modes for $\text{N}{{\text{O}}_{2}}^{-}$, with one nitrite bound in an asymmetric quasi-bidentate κ^2^-ONO manner and the other bound in a monodentate fashion with a linkage isomerism between the κ^1^-ONO and κ^1^-NO_2_ binding modes. We use density functional theory to help rationalize the presence of all three of these linkage isomers in one compound, before assessing the redox activity of [Cu**1**(NO_2_)_2_]. These latter studies show that the complex is not a competent nitrite reduction electrocatalyst in non-aqueous solvent, even in the presence of additional proton donors, a finding which may have implications for the design of biomimetic catalysts for nitrite reduction.

## 1. Introduction

The oxides of nitrogen are key constituents of the natural nitrogen cycle. Of these nitrogen oxides, nitrite ($\text{N}{{\text{O}}_{2}}^{-}$) occupies arguably the most prominent position, being an intermediate in a range of transformations between the various oxidation states of nitrogen [1]. The reduction of nitrite to nitric oxide (NO) is one such transformation, which is a critical step during the process of denitrification performed by a range of bacteria. The overall equation for this conversion is

1.1 $$\text{N}{{\text{O}}_{2}}^{-}+{\text{e}}^{-}+2{\text{H}}^{+}\to \text{NO}+{\text{H}}_{2}\text{O.}$$

Various classes of enzymes are involved in this transformation, based on haems, molybdenum and copper [2]. In the case of Cu, the enzymes performing this reaction are known as the copper-containing nitrite reductases (CuNIRs), and the reaction performed can therefore be written as

1.2 $$\text{Cu(I) }+\text{N}{{\text{O}}_{2}}^{-}+2{\text{H}}^{+}\to \text{Cu(II) }+\text{NO}+{\text{H}}_{2}\text{O.}$$

**Figure 1.**
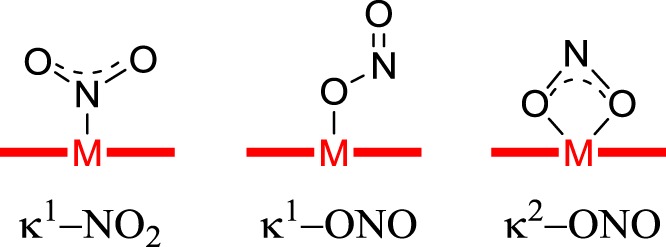
The modes of binding of nitrite to metal centres discussed in this paper.

The crystal structure of CuNIR (isolated from *Achromobacter cycloclastes*) was first reported by Adman and co-workers in 1991 [3]. This and subsequent studies have revealed that the active site for nitrite reduction consists of a Cu(II) ion coordinated by three histidine ligands and a single water molecule in a distorted tetrahedral geometry [4]. During nitrite reduction, $\text{N}{{\text{O}}_{2}}^{-}$ is believed to displace water from this complex and bind to the Cu(II) centre through both of its oxygen atoms (κ^2^-ONO binding mode; figure 1), leading to a distorted square-based pyramidal geometry at the Cu [2,5]. Subsequent electron transfer and protonation steps then lead to oxygen abstraction from the bound nitrite, NO liberation and the regeneration of the Cu(II) complex in its water-bound form, ready to enter the catalytic cycle again upon binding of additional nitrite [6,7].

The apparently rather simple active-site structure of CuNIR (a tripodal N-donor ligand which supports a copper centre undergoing a one-electron redox reaction) has led numerous groups of coordination chemists to attempt the synthesis of functional analogues of this enzyme [8–24]. Most of these complexes bind only one molecule of nitrite, but Woollard-Shore *et al.* [20] have reported two examples where two nitrite molecules are bound to a single Cu centre. In both of these examples, one nitrite is bound to the metal through both its oxygen atoms (κ^2^-ONO), while the other nitrite binds through a single oxygen (κ^1^-ONO). Lehnert and co-workers have also reported a series of copper complexes that bind nitrite in both a monodentate and a bidentate fashion (including a di-κ^1^-ONO complex and a κ^1^-ONO/κ^1^-NO_2_ complex) [25].

The discovery and investigation of more such binding mode isomers of tripodal N-donor copper complexes is important, both from the standpoint of understanding how the natural enzymes work and in terms of developing biomimetic copper catalysts for applications such as the removal of nitrite from water streams [26] and NO-release systems for biomedical applications [27]. However, to the best of our knowledge, no other examples of nitrite linkage isomerism in mononuclear copper complexes incorporating tripodal N-donor ligands have been reported to date, and no examples where nitrite is present in all three of its most common binding modes (κ^1^-NO_2_, κ^1^-ONO and κ^2^-ONO; figure 1) are known at all.

Herein, we report the synthesis and characterization of a new copper complex which contains a tripodal N-donor ligand (ligand **1**) and two molecules of nitrite. This complex ([Cu**1**(NO_2_)_2_]) forms immediately upon addition of nitrite to a solution of the precursor copper complex [Cu(**1**)_2_]^2+^, the synthesis and characterization of which we also report. [Cu(**1**)_2_]^2+^ shares a common geometry with a number of similar complexes reported in the 1990s by Jonas & Stack [28] although the effects of adding nitrite to these previously reported complexes have hitherto not been documented. [Cu**1**(NO_2_)_2_] (whose structure we determine by single crystal X-ray diffraction) displays all three common binding modes for $\text{N}{{\text{O}}_{2}}^{-}$, with one nitrite bound in an asymmetric quasi-bidentate κ^2^-ONO manner and the other bound in a monodentate fashion with linkage isomerism between κ^1^-ONO and κ^1^-NO_2_ binding modes (with approx. 0.62/0.38 occupancy, respectively). We use density functional theory (DFT) to help rationalize the presence of all three of these linkage isomers in one compound, before assessing the redox activity of [Cu**1**(NO_2_)_2_]. These latter studies show that the complex is not a competent nitrite electroreduction catalyst in non-aqueous solvent, even in the presence of additional proton donors, a finding which may have implications for the design of biomimetic catalysts for nitrite reduction.

## 2. Experimental section

### 2.1. General experimental remarks

Unless otherwise stated, all syntheses were conducted under nitrogen in air- and moisture-free solvents obtained from a commercial solvent purification system. Sodium hydride (60% dispersion in mineral oil), methyl iodide (99%), [Cu(CH_3_CN)_4_]PF_6_ (97%), benzoic acid (99.5%) and tetrabutylammonium nitrite (97%, TBA-NO_2_) were supplied by Sigma-Aldrich. Tetrabutylammonium hexafluorophosphate (TBA-PF_6_) (98%) was obtained from Alfa Aesar. 6-methyl-tris(2-pyridyl)methanol was prepared according to the published procedure [29].

All ^1^H and ^13^C NMR spectra were recorded on a Bruker AV 400 instrument, at a constant temperature of 300 K. Chemical shifts are reported in parts per million from low to high field. Coupling constants (*J*) are reported in hertz (Hz). Standard abbreviations indicating multiplicity were used as follows: m, multiplet; d, doublet; s, singlet. UV–vis spectra were recorded on a JASCO V-670 spectrophotometer using 1 cm path length cuvettes. CHN analyses were collected by the services facility at the School of Chemistry, University of Glasgow, as were LM-MS mass spectra (ESI, positive mode, Bruker micrOTOF-Q machine). IR spectra were collected in the solid state on a Shimadzu IRAffinity-1S Fourier Transform Infrared Spectrophotometer. X-band electron paramagnetic resonance (EPR) spectra were recorded on a Bruker ELEXSYS E500 Spectrometer and simulations were performed using Bruker's Xsophe Program Package [30]. Melting points were gauged using an Electrothermal IA 9000 digital melting point machine. Experiments performed at ‘room temperature' were carried out at 20°C. Electrochemical experiments were performed as below.

### 2.2. Synthesis of ligand **1**

**Scheme 1.**
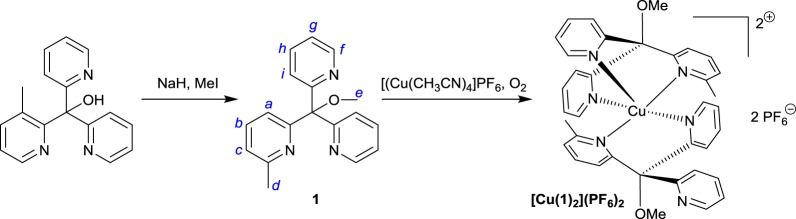
The general synthetic route followed to synthesize ligand **1** and complex [Cu(**1**)_2_](PF_6_)_2_. Italic letters on the product structures correspond to the ^1^H NMR signal assignments in the ‘Experimental section’.

6-methyl-tris(2-pyridyl)methanol (0.380 g, 1.37 mmol, 1 eq.) was dissolved in THF (7 ml) under nitrogen. Meanwhile, a suspension of NaH (60% dispersion in mineral oil, 0.274 g, 6.85 mmol, 5 eq.) was prepared in 25 ml THF under nitrogen. To this suspension, the solution of 6-methyl-tris(2-pyridyl)methanol was then added via cannula, followed by 0.43 ml (0.972 g, 6.85 mmol, 5 eq.) of methyl iodide to give an off-white suspension. The reaction mixture was then heated up to 60°C under nitrogen overnight, during which time it was observed to turn orange. After cooling to room temperature, the reaction mixture was diluted with 100 ml CHCl_3_ and then washed with water and then brine. The organic phase was dried over MgSO_4_ to give a clear orange solution, which was concentrated *in vacuo* to yield a reddish-orange oil. To this oil was added 25 ml acetonitrile and the resulting suspension was extracted with 25 ml of pentane, whereupon two clear phases became evident: an upper colourless (pentane) layer and a lower bright yellow (acetonitrile) layer. Collection of the acetonitrile layer after further washing with pentane (2 × 25 ml) and concentration under reduced pressure then yielded compound **1** as an off-white solid (0.373 g, 93%), m.p. = 101–103°C. Anal. calcd. for C_18_H_17_N_3_O: C 74.20, H 5.88, N 14.42. Found: C 73.80, H 5.89, N 14.13. ^1^H NMR (CDCl_3_, 400 MHz), *δ* = 8.51–8.49 (m, 2H, H_f_), 7.64 (dt, 2H, *J_1_* *=* 7.8, *J_2_* *=* 1.1, H_i_), 7.58 (td, 2H, *J_1_* *=* 7.2, *J_2_* *=* 1.6, H_g_ or H_h_), 7.47 (t, 1H, *J* *=* 7.6, H_b_), 7.38 (d, 1H, *J* *=* 7.6, H_a_ or H_c_), 7.09–7.05 (m, 2H, H_g_ or H_h_), 6.93 (d, 1H, *J* *=* 7.6, H_a_ or H_c_), 3.21 (s, 3H, H_e_), 2.40 (s, 3H, H_d_). Assignments of signals to groups of protons are based on two-dimensional (COSY) spectra and the expected coupling patterns of the peaks, although the symmetry of the molecule prevents unambiguous assignments. Letter codes correspond to those shown in scheme 1. The ^1^H NMR spectrum of this compound is shown in electronic supplementary material, figures S1 and S2. ^13^C NMR (CDCl_3_, 100 MHz), *δ* = 161.8, 160.6, 157.2, 148.4, 136.0, 135.8, 124.1, 121.8, 121.5, 120.8, 88.5, 53.0, 24.7. The ^13^C NMR spectrum of this compound is shown in electronic supplementary material, figure S3. ESI-LMMS (methanol): *m/z* = 314.1257 [M + Na]^+^ (calcd. for C_18_H_17_N_3_NaO; 314.1269). IR (solid state, cm^−1^) *ν* = 3066 (br), 2980 (w), 2956 (w), 2927 (m), 1583 (s), 1572 (s), 1461 (s), 1446 (s), 1435 (s), 1113 (s), 1081 (s), 985 (s), 802 (s), 771 (s), 751 (s).

### 2.3. Synthesis of complex [Cu(**1**)_2_](PF_6_)_2_

Free ligand **1** (0.260 g, 0.89 mmol, 1 eq.) was dissolved in acetonitrile (10 ml) under nitrogen. Meanwhile, a solution of [Cu(CH_3_CN)_4_]PF_6_ (0.316 g, 0.85 mmol, 0.95 eq.) was prepared in 50 ml of acetonitrile under nitrogen. The solution of compound **1** was then added to the solution of the copper salt dropwise over 30 min at room temperature, producing a bright yellow solution. After complete addition, the reaction mixture was stirred for a further 90 min at room temperature, resulting in a yellow solution and a white precipitate. The solution was then filtered under air, and the volume of the filtrate reduced by half. Diethyl ether was then added to the filtrate. This yellow solution was subsequently sealed to prevent evaporation of the solvents and allowed to stand at room temperature under air for two weeks. After this time, the reaction mixture was observed to consist of a lilac solution and a blue-green precipitate. The precipitate was removed by filtration and the purple filtrate concentrated *in vacuo* to yield [Cu(**1**)_2_](PF_6_)_2_ as a lilac solid (0.325 g, 80% based on ligand **1**), m.p. = 200°C (dec.). Anal. calcd. for C_36_H_34_CuF_12_N_6_O_2_P_2_: C 46.19, H 3.66, N 8.98. Found: C 46.35, H 3.63, N 9.05. ESI-LMMS (methanol): *m/z* = 354.0589 [Cu + **1**]^+^ (calcd. for C_18_H_17_CuN_3_O; 354.0668); *m/z* = 322.5938 [Cu + (**1**)_2_]^2+^ (calcd. for C_36_H_34_CuN_6_O_2_; 322.6020). IR (solid state, cm^−1^) *ν* = 3115 (vw, br), 2987 (vw, br), 1599 (m), 1573 (w), 1474 (m), 1451 (m), 1433 (m), 1092 (m), 830 (s), 762 (m). Absorption spectrum (acetonitrile): *λ*_max_, *ε*; 560 nm, 56 M^−1^ cm^−1^.

### 2.4. Synthesis of complex [Cu**1**(NO_2_)_2_]

To a solution of [Cu(**1**)_2_](PF_6_)_2_ (0.065 g, 0.069 mmol, 1 eq.) in acetonitrile (1 ml) was added a solution of tetrabutylammonium nitrite (0.040 g, 0.14 mmol, 2 eq.) in acetonitrile (0.5 ml), giving a dark green solution. After agitation for 5 min at room temperature, 12 ml of diethyl ether were added to the reaction vessel, which was then sealed and stored at 4°C overnight. In the morning, large (1–2 mm) green, column-shaped crystals were obtained, suitable for X-ray diffraction (see below). These crystals were then washed with diethyl ether and dried at 120°C, giving [Cu**1**(NO_2_)_2_] as dark green crystals (0.022 g, 71%), m.p. = 215°C (dec.). Anal. calcd. for C_18_H_17_CuN_5_O_5_: C 48.38, H 3.83, N 15.67. Found: C 48.40, H 3.73, N 15.12. ESI-LMMS (acetonitrile): *m/z* = 354.0564 [Cu + **1**]^+^ (calcd. for C_18_H_17_CuN_3_O; 354.0668). IR (solid state, cm^−1^) *ν* = 3117 (br), 1597 (s), 1451 (m), 1437 (m), 1373 (s), 1327 (m), 1128 (s), 1087 (s), 1025 (m), 772 (s), 657 (s). Absorption spectrum (acetonitrile): *λ*_max_, *ε*; 643 nm, 120 M^−1^ cm^−1^.

### 2.5. Electrochemical methods

Electrochemical studies were performed in a single-chamber cell with a three-electrode configuration using a CH Instruments CHI700 series potentiostat. The supporting electrolyte was 0.1 M TBA-PF_6_ in acetonitrile. A large surface area strip of carbon felt (Alfa Aesar) was used as the counter electrode, along with an Ag/AgNO_3_ pseudo-reference electrode. Potentials are reported relative to the ferrocenium/ferrocene couple, the position of which was judged by adding ferrocene to the samples analysed. Working electrodes were washed with acetone and deionized water prior to use. Cyclic voltammograms were collected at room temperature under an atmosphere of Ar at a scan rate of 100 mV s^−1^. A glassy carbon disc electrode (area = 0.071 cm^2^, CH Instruments) was used as the working electrode for cyclic voltammetry. Measurements were conducted without stirring and with *i*R compensation enabled.

### 2.6. Computational methods

All calculations were performed with the program Gaussian 09 [31] at the DFT level. The M06-L exchange–correlation functional was used, [32] which is a pure functional of the meta-GGA type, which has been shown to perform well for transition-metal complexes [33,34]. The def2-TZVP basis set was used, [35] with the ‘W06’ auxiliary basis for density fitting [36]. The effect of exact exchange was gauged by comparing to the M06 functional, [37] which is a hybrid meta-GGA with 27% exact-exchange admixture. No significant differences in structures, energies or spin density distributions were found. Interestingly, however, vibrational frequencies calculated with M06 were blue-shifted by as much as 80 cm^–1^ compared with M06-L. Modes involving N–O stretching were particularly strongly affected. The agreement with experiment was significantly better for the M06-L frequencies. All computational results reported in the paper were therefore obtained with M06-L/def2-TZVP; data obtained with M06 are available *via* the University of Glasgow's open access online data repository [38]. All structures were fully optimized; stationary points were identified as minima by the absence of imaginary frequencies. Gibbs free energies were calculated based on the usual harmonic-oscillator/rigid-rotor/ideal-gas approximation at 298.15 K and 101.325 kPa. Structures were manipulated and visualized with the programs Avogadro [39,40], Jmol [41] and ChemCraft [42].

### 2.7. Crystallography

Crystallographic data were collected at the University of Glasgow on a Bruker APEX-II CCD diffractometer. A green, column-shaped crystal of dimensions 0.46 × 0.1 × 0.06 mm was used for single crystal X-ray diffraction data collection. C_18_H_17_CuN_5_O_5_ crystallized in the triclinic space group *P − *1 (space group No. 2), with unit cell dimensions *a* = 8.7123 (17), *b* = 8.7388 (17), *c* = 14.342 (3), *α* = 72.586 (4)°, *β* = 73.955 (4)°, *γ* = 62.928 (4)° and *V* = 915.0 (3) Å^3^, *T* = 100 K. In total, 15 440 reflections were measured by *ω* scans, with 3197 independent reflections with *R*_int_ = 0.079, *θ*_max_ = 25.0°, *θ*_min_ = 1.5° using Mo *Kα* radiation, *λ* = 0.71073 Å. The structure was solved using SHELXS and refined using SHELXL (both within OLEX2) [43,44]. OLEX2 was also used for molecular graphics and to prepare material for publication. CCDC 1547352 contains the supplementary crystallographic data for this paper. More details on the crystallographic data and its collection can be found in the electronic supplementary material.

## 3. Results and discussion

### 3.1. Synthesis of ligand **1** and complex [Cu(**1**)_2_](PF_6_)_2_

A general route to the synthesis of ligand **1** and copper complex [Cu(**1**)_2_](PF_6_)_2_ is given in scheme 1. Hence, deprotonation of 6-methyl-tris(2-pyridyl)methanol [29] with NaH followed by methylation with methyl iodide yielded the free ligand (**1**) in excellent yield after purification. Compound **1** was methylated in this way in order to prevent any coordination of the alcohol group to the metal centre, which it was feared might interfere in the subsequent binding of the metal centre to any small molecule ligands that were subsequently added.

**Figure 2.**
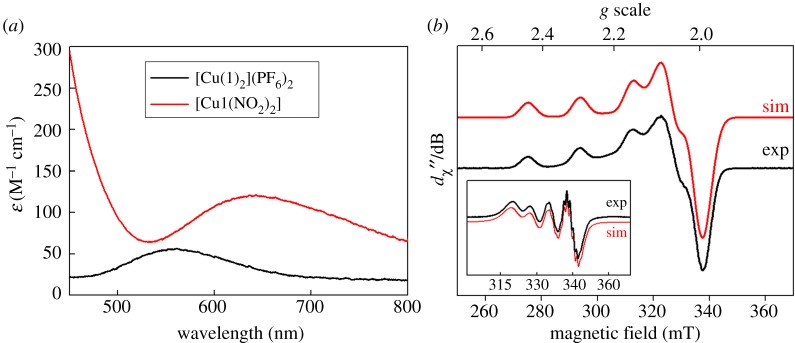
(*a*) UV–vis spectra of complex [Cu(**1**)_2_](PF_6_)_2_ in acetonitrile (black line) and of this complex after addition of 10 equivalents of TBA-NO_2_, yielding [Cu**1**(NO_2_)_2_] (red line). (*b*) The X-band EPR spectrum of [Cu(**1**)_2_](PF_6_)_2_ recorded in acetonitrile/dichloromethane solution at 130 K (conditions: frequency, 9.4201 GHz; power, 2.0 mW; modulation, 0.2 mT). Experimental data are represented by the black line and the simulation is depicted by the red trace; simulation parameters are given in the text. The inset shows the fluid solution spectrum at 293 K (experimental conditions: frequency, 9.854 GHz; power, 10 mW; modulation, 0.5 mT).

After methylation, copper was then inserted into ligand **1** by stirring the free ligand with [Cu(CH_3_CN)_4_]PF_6_ at room temperature. Subsequent exposure to air then yielded the purple complex [Cu(**1**)_2_](PF_6_)_2_. The electronic absorption spectrum of this complex (figure 2*a*, black line) showed strong similarities to that reported for the analogous complex (containing an -OMe substituent on the pyridyl ring in place of the methyl substituent) reported previously by Jonas & Stack ([Cu(**1**)_2_]^2+^ displays *λ*_max_ at 560 nm, with an extinction coefficient of 56 M^−1^ cm^−1^, while the -OMe derivative has *λ*_max_ = 580 nm with *ε* = 57 M^−1^ cm^−1^) [28]. The position of the absorption maximum for this transition at a value well below 600 nm was previously rationalized on the basis of the Jahn–Teller distortion which is expected for a d^9^ Cu(II) ion ligated by six almost equivalent ligands. This distortion, it was argued, should lead to ligand field transitions at higher energies as the distortion away from octahedral geometry becomes greater (and as the Cu−N bonds in the equatorial plane therefore become shorter). A *λ*_max_ value of 560 nm therefore corresponds to a complex in which the geometry is significantly distorted away from octahedral symmetry. To obtain further insight into the nature of the geometry around the copper centre in [Cu(**1**)_2_]^2+^, EPR spectroscopy was performed on [Cu(**1**)_2_](PF_6_)_2_. The fluid solution spectrum in acetonitrile (inset to figure 2*b*) is centred at *g*_iso_ = 2.109 and exhibits the classic four-line signal for Cu(II), with a coupling of 71.4 × 10^–4^ cm^–1^ to the ^63,65^Cu isotopes (*I* = ^3^/_2_; 100% abundant). At the high-field end of the spectrum, where molecular tumbling affords the narrowest linewidth, additional hyperfine splittings are observed. The spectrum was well reproduced by including a superhyperfine coupling of 13.1 × 10^–4^ cm^–1^ to four ^14^N nuclei (*I* = 1, 99.7% abundant)—two from each of the tripodal ligands that constitute the donor atoms to the singly occupied d*_x_*_2−*y*2_ orbital.

A frozen solution spectrum was recorded in an acetonitrile/dichloromethane glass at 130 K (figure 2*b*, main panel). This has the signature axial profile of a Cu(II) d^9^ ion with a ^2^B_1_ ground state, with *g*_||_ > *g*_⊥_ > *g*_e_ (free electron *g*-value) and *A*_||_ ≫ *A*_⊥_. The simulation gave spin-Hamiltonian parameters: *g* = (2.057, 2.058, 2.227); *A*{^63,65^Cu} = (10, 10, 193) × 10^–4^ cm^–1^; *A*{^14^N} = (13, 13, 12) × 10^–4^ cm^–1^. Although the superhyperfine coupling from the ^14^N nuclei was less than the experimental linewidth, it was nonetheless included and approximated by matching the average value to the one obtained from the isotropic spectrum. There is a slight drift away from strict axial symmetry, with the best fit obtained with inequivalent *g_x_* and *g_y_* values. This stems from the methylation of one pyridyl arm in **1**. The spectral profile is near-identical to that reported by Jonas & Stack [28] for their analogous complex with methoxylated pyridyl groups, and is typical of complexes of Cu(II) in five-coordinate and distorted octahedral geometries with pyridyl ligands [45–47].

### 3.2. Synthesis and structure of complex [Cu**1**(NO_2_)_2_]

**Figure 3.**
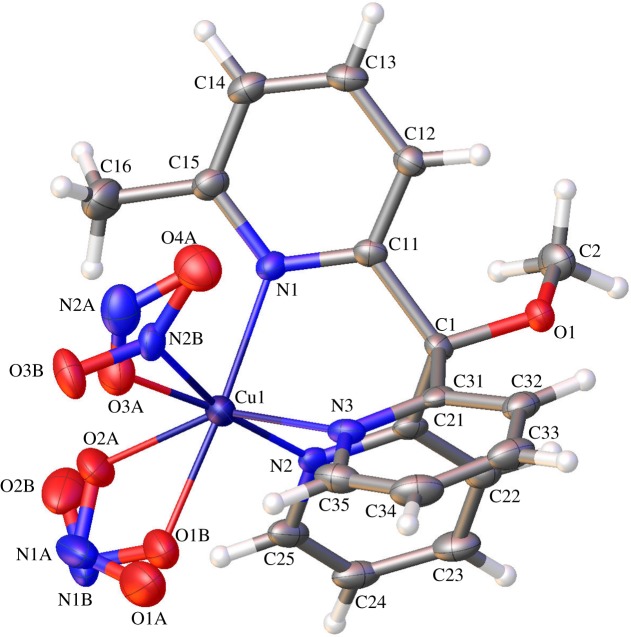
The crystal structure of [Cu**1**(NO_2_)_2_]. Crystallographic details can be found in the electronic supplementary material. Colour scheme: C, grey; N, blue; O, red; Cu, purple and H, white.

Upon addition of nitrite to [Cu(**1**)_2_](PF_6_)_2_, the complex undergoes rapid reaction as signalled by an immediate colour change from lilac to bottle-green. Hence, when 10 equivalents of tetrabutylammonium nitrite (TBA-NO_2_) were added to a 1.2 mM solution of [Cu(**1**)_2_](PF_6_)_2_ in acetonitrile, the colour change was instantaneous and gave rise to the absorption spectrum shown as the red line in figure 2*a* (*λ*_max_ = 643 nm, *ε* = 120 M^−1^ cm^−1^). This reaction was repeated on a preparative scale, allowing green crystals of [Cu**1**(NO_2_)_2_] to be obtained by vapour diffusion of diethyl ether into an acetonitrile solution of [Cu**1**(NO_2_)_2_]. These crystals then proved suitable for X-ray diffraction (figure 3).

The crystal structure of [Cu**1**(NO_2_)_2_] shows the copper centre to be coordinated octahedrally, with three Cu−N bonds to the pyridine moieties in ligand **1** and a further three bonds between the Cu centre and two nitrite anions. The Cu–N bond lengths between the Cu and the nitrogen donors in ligand **1** are: Cu–N1 = 2.122(3), Cu–N2 = 2.009(3) and Cu–N3 = 2.169(4) Å, which are in reasonable agreement with the distances reported previously by Tanaka and co-workers for Cu(II)-tris(2-pyridyl) complexes with only one bound nitrite [48,49] and Kodera *et al.* [50] for Cu(II)-tris(2-pyridyl) complexes featuring two bound bromides.

More interestingly, the structure also displays isomerism in the two bound nitrite ligands. The first nitrite (defined by nitrogen N1A/N1B and oxygens O1A/O1B and O2A/O2B) is bound to the Cu(II) centre in an asymmetric quasi-bidentate manner with some disorder in the bond lengths, reminiscent of that observed by Lehnert and co-workers [25]. This disorder comes about as a result of the two Cu−O bond lengths being slightly different in the two isomers, which have N1A/B occupancies in a ratio of approximately 0.55 : 0.45. For example, the Cu−O1B bond length is 2.010(18) Å, while the Cu−O2B distance is considerably longer at 2.66(2) Å. Similar quasi-bidentate interactions of nitrite with a Cu(II) centre in a tris(2-pyridyl) complex have previously been reported by Woollard-Shore *et al*. [20] who found a long Cu−O interaction of 2.5620 Å.

**Figure 4.**
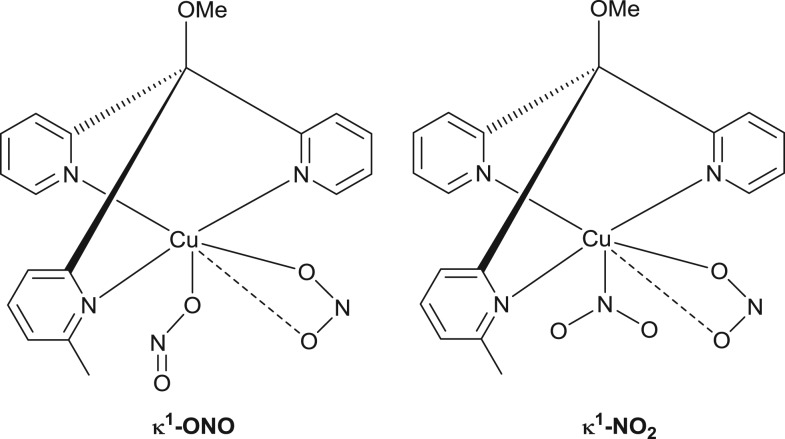
Chemical structures of the linkage isomers observed by single crystal X-ray diffraction of [Cu**1**(NO_2_)_2_]. These structures are based on the crystallographic data summarized in figure 3.

The second nitrite in the structure (defined by nitrogen N2A/N2B and oxygens O3A/O3B and O4A) is bound in a monodentate fashion. Again, however, there is disorder in the nature of this binding, this time a linkage isomerization between κ^1^-ONO and κ^1^-NO_2_ binding modes (with approx. 0.62/0.38 occupancy, respectively). The structure of the O-bound isomer is shown on the left-hand side of figure 4. Such a combination of κ^1^-ONO and quasi-κ^2^-ONO binding modes for nitrite ligands on Cu(II)-tris(2-pyridyl) complexes has been reported before by Woollard-Shore *et al*. [20]. In our case, the coordinating oxygen (O3A) displays a Cu−O3A bond length of 1.922(5) Å and unequal N−O bond lengths within the κ^1^-bound nitrite of O3A–N2A = 1.227(8) Å and O4A–N2A = 1.171(7) Å. These compare to a Cu−O coordination bond of length 1.9701(17) Å and internal N−O bond lengths of 1.290(3) and 1.221(3)Å in the previously reported structure [20].

The second linkage isomer (see the κ^1^-NO_2_ structure on the right-hand side of figure 4) evinces a Cu1−N2B bond length of 2.147(14) Å and more equal internal bond lengths (O3B−N2B = 1.249(12) and O4A−N2B = 1.276(12) Å) when compared with the κ^1^-ONO linkage isomer. The presence of both quasi-κ^2^-bound and κ^1^-bound nitrite isomers, together with further isomerism in the linkage of the κ^1^-bound isomers is, to the best of our knowledge, unprecedented for copper complexes (although Raithby and co-workers have reported such behaviour for nickel(II) complexes with tripodal *N,N,N*-donor ligands [51–54]).

To clarify the energetic and electronic aspects of the two types of isomerism and to analyse the structures of the different forms, we undertook a series of DFT calculations. There are four isomeric forms to consider: both of the κ^1^-bound linkage isomers (κ^1^-ONO and κ^1^-NO_2_) can exist in two forms, depending on which of the Cu–O bonds of the quasi-chelating κ^2^-ONO ligand is short or long. Because these two forms are de facto enantiomeric (but for the methyl groups, which are remote from the metal centre), they are energetically degenerate for all intents and purposes, which is consistent with the approximately 1 : 1 occupancy in the X-ray structure. It is, therefore, sufficient to consider in the following only one form of each of these linkage isomers.

**Figure 5.**
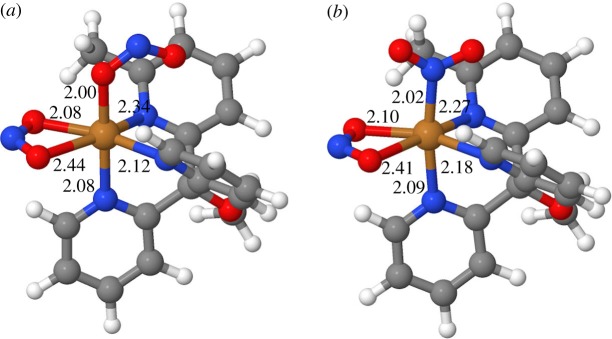
DFT-optimized structures of the two linkage isomers of [Cu**1**(NO_2_)_2_], κ^2^-ONO, κ^1^-ONO (*a*) and κ^2^-ONO, κ^1^-NO_2_ (*b*). Selected metal–donor distances are given in Å.

As can be seen from figure 5, the corresponding copper–donor distances for the two linkage isomers are very similar. The quasi-chelating κ^2^-ONO ligand is bound asymmetrically, with one short and one long Cu–O interaction, as seen in the X-ray structure. This asymmetry in the coordination is mirrored in the Cu–N_pyr_ distances *trans* to the bidentate nitrite: the two short Cu–N/O bonds are (approx.) *trans* to each other, as are the two long ones. This feature is not apparent from the X-ray structure, where these Cu–N_pyr_ bonds have similar lengths (2.12 and 2.17 Å) and the nitrogens have unique positions. Such asymmetry in the coordination is expected for an octahedral d^9^ system: the two long Cu–N/O bonds define the Jahn–Teller axis, along which the octahedron is tetragonally distorted (elongated).

**Figure 6.**
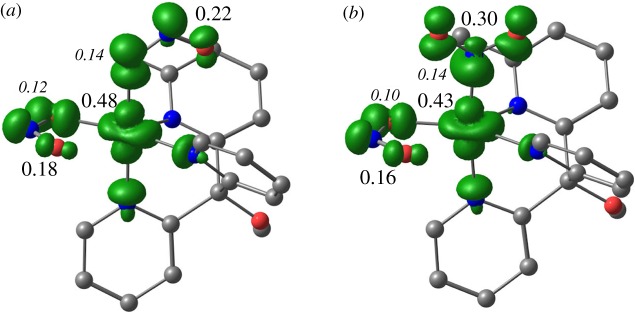
Net spin density isosurfaces (*ρ* = 0.005) for the two linkage isomers of [Cu**1**(NO_2_)_2_], κ^2^-ONO, κ^1^-ONO (*a*) and κ^2^-ONO, κ^1^-NO_2_ (*b*). Selected Mulliken spin populations are given in units of electron spin; large, Roman labels refer to Cu and the nitrite ligands as a whole; smaller, italic labels refer to single donor atoms. Hydrogens have been omitted for clarity.

Looking at the spin density distributions and Mulliken spin populations for the two linkage isomers (figure 6), there is again very little difference between them. In essence, around half of the spin of one electron is localized on the metal and approximately 0.2 of the spin of one electron is localized on each of the nitrite ligands. The nitrites therefore carry a significant fraction of the unpaired spin density. The shape of the spin density on copper is clearly recognizable as that corresponding to the ${\text{d}}_{x^{2}-y^{2}}$ orbital, which is the SOMO in these complexes (noting that the Jahn–Teller axis is taken to be the *z*-axis by definition).

The two linkage isomers are nearly energetically degenerate. The κ^2^-ONO, κ^1^-NO_2_ isomer is calculated to be less stable than the κ^2^-ONO, κ^1^-ONO isomer by 4 kJ mol^–1^ in terms of potential energy and just 1 kJ mol^–1^ in terms of Gibbs free energy. The small energy difference is consistent with the concurrent presence of both isomers in the crystal in approximately equal proportion. Similarly, small energy differences between O- and N-coordinated nitrite isomers have been reported before by Lehnert and co-workers for their series of Cu complexes [25].

**Figure 7.**
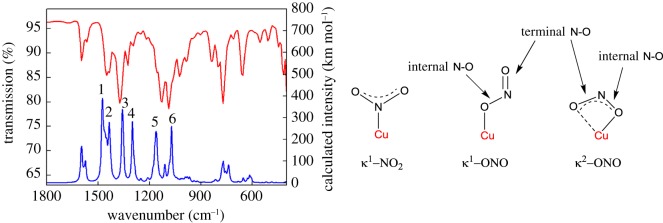
Experimental (red line) and calculated (blue line) infrared spectra of [Cu**1**(NO_2_)_2_] and a depiction of the various N–O stretching modes that are possible. Number labels on the calculated spectrum correspond to the stretches discussed in the main text below (the calculated spectrum is the sum of the spectra for the two linkage isomers and has been shifted by –40 cm^–1^). See electronic supplementary material, figure S4 for deconvoluted calculated spectra for the two linkage isomers.

The infrared spectrum of [Cu**1**(NO_2_)_2_] reveals a number of peaks that fall in the frequency range characteristic of bound nitrite. However, the assignment of these is complicated by the fact that several of these bands (bands 1, 2 and 6 in figure 7) overlap with bands exhibited by ligand **1** and [Cu(**1**)_2_](PF_6_)_2_ (see ‘Experimental section’). Hence, only the bands at 1373, 1327 and 1131 cm^−1^ (labels 3, 4 and 5, respectively) are present in the infrared spectrum of [Cu**1**(NO_2_)_2_] but not in the spectra of ligand **1** and [Cu(**1**)_2_](PF_6_)_2_.

Based on the literature, two alternatives then exist for the assignment of these bands. In the first scenario, the bands at 1373 and 1131 cm^−1^ emanate from terminal and internal N–O stretches of O-bound nitrite, respectively [48]. Meanwhile, the band at 1327 cm^−1^ is assigned to the κ^1^-NO_2_ symmetric stretching mode [20]. In the second scenario, the two bands at 1373 and 1327 cm^−1^ are assigned to terminal N–O stretches from O-bound nitrites, and now the band at 1131 cm^−1^ is assigned to the κ^1^-NO_2_ symmetric stretching mode [25]. Our calculated vibrational data suggest that perhaps the first scenario is the more likely. Taking into account that the M06-L functional somewhat overestimates vibrational frequencies (especially modes involving N–O stretching), we predict that the κ^1^-NO_2_ symmetric stretch should appear between 1340 and 1300 cm^−1^. Furthermore, we predict that a terminal N–O stretch from κ^2^O-bound nitrite should manifest between 1400 and 1354 cm^−1^, with an internal N–O stretch from this O-bound nitrite featuring between 1203 and 1158 cm^−1^. Regardless, however, of which of these scenarios is more correct, both scenarios agree that nitrite is present in [Cu**1**(NO_2_)_2_] in both N-bound and O-bound forms, in agreement with the X-ray diffraction data.

**Figure 8.**
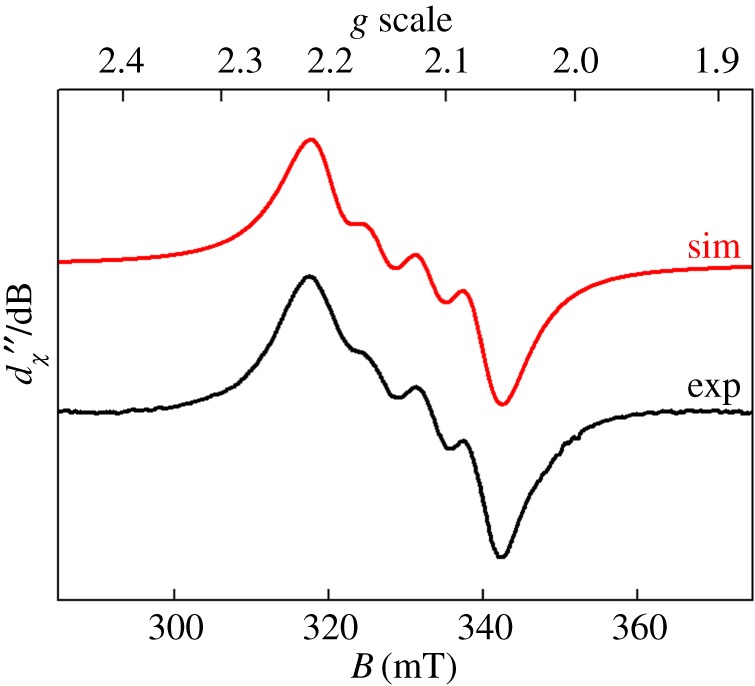
X-band EPR spectrum of [Cu**1**(NO_2_)_2_] recorded in acetonitrile solution at 293 K (experimental conditions: frequency, 9.854 GHz; power, 10 mW; modulation, 0.5 mT). Experimental data are represented by the black line; the simulation is depicted by the red trace.

The EPR spectrum of [Cu**1**(NO_2_)_2_] in acetonitrile (figure 8) showed a shift of the resonant field position to the lower field and a concomitant decrease in the ^63,65^Cu hyperfine coupling constant. The line broadening compared with [Cu(**1**)_2_](PF_6_)_2_ is a consequence of perturbed molecular tumbling relative to the timescale of the experiment and therefore any superhyperfine features indicating the N-donor ligands in the first coordination sphere were obscured. The spectrum was simulated with *g*_iso_ = 2.132 and *A*_iso_ = 63 × 10^–4^ cm^–1^. The smaller Cu hyperfine coupling reveals less spin density localized on the Cu centre, consistent with a six-coordinate complex. A frozen solution spectrum of this complex recorded in an acetonitrile/dichloromethane glass at 130 K is shown in the electronic supplementary material, figure S5.

### 3.3. Redox activity of [Cu**1**(NO_2_)_2_] and nitrite electroreduction studies

**Figure 9.**
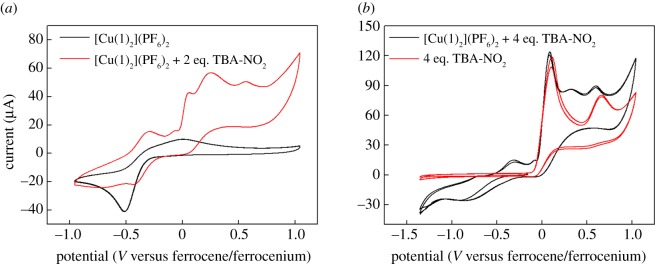
Cyclic voltammograms of a 2.8 mM acetonitrile solution of complex [Cu(**1**)_2_]^2+^ containing 0.1 M TBA-PF_6_ and various amounts of TBA-NO_2_ run under the conditions detailed in the ‘Experimental section’. (*a*) The black trace shows the behaviour of [Cu(**1**)_2_]^2+^ on its own, and the red trace that of [Cu(**1**)_2_]^2+^ in the presence of 2 equivalents of TBA-NO_2_. (*b*) The black trace shows the behaviour of [Cu(**1**)_2_]^2+^ in the presence of 4 equivalents of TBA-NO_2_, and the red trace that of the equivalent amount of TBA-NO_2_ on its own.

On account of the known nitrite electroreduction activity of allied Cu-tris(2-pyridyl) complexes [22,23,48], we hypothesized that [Cu**1**(NO_2_)_2_] might also be active for this transformation. Initially, cyclic voltammetry was performed on a nitrite-free solution of [Cu(**1**)_2_](PF_6_)_2_ in acetonitrile containing 0.1 M tetrabutylammonium hexafluorophosphate (TBA-PF_6_) as the supporting electrolyte. This evinced a reduction process centred at around −0.5 V (versus the ferrocenium/ferrocene couple), which we attribute to reduction of the Cu(II) centre to the Cu(I) oxidation state by analogy to previous studies of such complexes in non-aqueous solvents (black trace in figure 9*a*) [28]. However, this redox event is only quasi-reversible, with the re-oxidation event occurring over a rather broad range and with considerable separation between the cathodic and anodic peaks. Such behaviour has been noted previously for complexes consisting of two tripodal ligands coordinated to one Cu(II) centre [28] and would be consistent with extensive ligand rearrangements taking place as a result of reduction and re-oxidation of the copper centre. Hence, reduction of the Cu(II) centre in [Cu(**1**)_2_]^2+^ would produce a Cu(I) centre, which would be likely to exist preferentially in a tetrahedral coordination environment. Hence, loss of one of the bound tripodal ligands may occur or both of the tripodal ligands may remain bound but undergo significant rearrangements as a result of reduction. Re-oxidation would then require a change in geometry back to a higher coordination number. Such a scenario would also be consistent with the reproducible nature of the CVs of [Cu(**1**)_2_]^2+^ under these conditions: repeated cycling over the range shown by the black trace in figure 9*a* does not lead to any decrease in the signal or alteration in the shape of the voltammogram, consistent with slow ligand rearrangements that accommodate the preferred coordination geometry of these two oxidation states of copper [28].

Upon addition of two equivalents of a soluble nitrite source to [Cu(**1**)_2_]^2+^, the colour of the solution turns from lilac to green (indicating the formation of [Cu**1**(NO_2_)_2_]) and the redox wave attributed to the Cu(II)/Cu(I) couple appears to shift to slightly more anodic potentials, as shown by the red trace in figure 9*a*. Upon addition of excess nitrite, the Cu(II)/Cu(I) couple becomes less well defined (black trace in figure 9*b*). Meanwhile, the oxidation events that were evident upon the addition of two equivalents of nitrite (at 0 V and above) become more pronounced. However, these oxidation events seem to be due mainly to the background oxidation of nitrite as indicated by the red trace in figure 9*b*, which shows that these waves are present when the nitrite salt is dissolved in the electrolyte in the absence of any copper complex.

**Figure 10.**
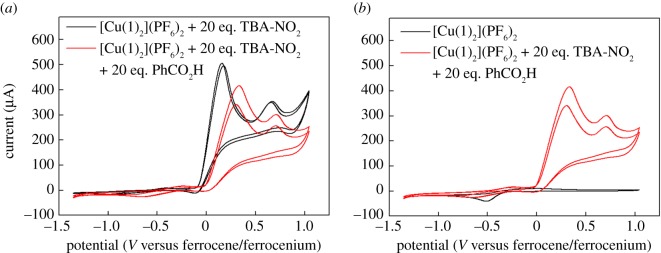
Cyclic voltammograms of a 2.8 mM acetonitrile solution of complex [Cu(**1**)_2_]^2+^ containing 0.1 M TBA-PF_6_ and various amounts of TBA-NO_2_ run under the conditions detailed in the ‘Experimental section’. (*a*) The black trace shows the behaviour of [Cu(**1**)_2_]^2+^ in the presence of 20 equivalents of TBA-NO_2_, and the red trace that of [Cu(**1**)_2_]^2+^ in the presence of 20 equivalents of TBA-NO_2_ and 20 equivalents of benzoic acid. (*b*) The black trace shows the behaviour of [Cu(**1**)_2_]^2+^ on its own, and the red trace that of [Cu(**1**)_2_]^2+^ in the presence of 20 equivalents of TBA-NO_2_ and 20 equivalents of benzoic acid.

Further additions of nitrite to solutions of [Cu**1**(NO_2_)_2_] show an increase in the wave corresponding to background nitrite oxidation, but do not show any catalytic waves that could be attributed to the reduction of nitrite (figure 10*a*, black trace). Hence, we concluded that no electrocatalytic reduction of nitrite as a result of the presence of our copper complex was occurring under these conditions. This is perhaps not unexpected, as equations (1.1) and (1.2) clearly show that the most probable nitrite reduction reactions all require protons. We therefore attempted to switch on electrocatalysis in our system by the addition of a soluble proton source (in this case, benzoic acid). Tripodal copper complexes have previously been used to demonstrate nitrite electroreduction to NO in aqueous solution, and these studies have shown that nitrite reduction does not occur at pH values above 9, on account of the dearth of protons [22,23,48]. There was, therefore, good reason to suppose that addition of an external proton source would be efficacious in our case. However, the red trace in figure 10*a* shows that adding 20 equivalents of benzoic acid to a solution containing 20 equivalents of nitrite and one equivalent of copper complex fails to produce any reductive features that could be interpreted as a catalytic wave. Indeed, figure 10*b* shows an overlay of the red trace from figure 10*a* with the CV displayed by the [Cu(**1**)_2_]^2+^ ion on its own, without any nitrite or benzoic acid present. This clearly shows that there is no reductive activity in the presence of nitrite and benzoic acid that exceeds in magnitude the current for the reduction of the complex alone. On this basis, we are forced to conclude that [Cu**1**(NO_2_)_2_] is not an active electrocatalyst for nitrite reduction under these conditions, even in the presence of an external proton source.

## 4. Conclusion

In summary, we have reported the synthesis and characterization of the first example of a copper complex ([Cu**1**(NO_2_)_2_]) containing all three of the most common binding modes for nitrite (κ^2^-ONO, κ^1^-ONO and κ^1^-NO_2_), as confirmed by single crystal X-ray diffraction. Furthermore, the diffraction data indicate that the relative populations of species containing κ^1^-ONO and κ^1^-NO_2_ linkages are around 0.62 and 0.38, respectively. Using DFT, we then rationalize the appearance of both of these linkage isomers in the same compound on the basis of the very small difference in energy between these two forms. The solution-phase behaviour of [Cu**1**(NO_2_)_2_] (probed by EPR and UV–vis spectroscopy) also suggests that the copper centre is bound to two nitrites. However, electrochemical studies reveal that [Cu**1**(NO_2_)_2_] is not a competent nitrite reduction electrocatalyst in acetonitrile, even in the presence of additional proton donors. This was a surprise to us, given that analogous complexes have been reported to perform this transformation in aqueous environments. The lack of reactivity in our case therefore warrants further investigation, with the inclusion on the tripodal ligand of pendant acid–base units that can mediate proton-coupled-electron transfer [55] being one such avenue that we will explore.

## Supplementary Material

Symes_Supplementary information_ESM.doc

## Data accessibility

The data which underpin this work are available via Enlighten, the University of Glasgow's open access online data repository, at http://dx.doi.org/10.5525/gla.researchdata.416. CCDC 1547352 contains the supplementary crystallographic data for this paper. These data can be obtained free of charge from The Cambridge Crystallographic Data Centre via www.ccdc.cam.ac.uk/data_request/cif.
