# Supplementary material for: An investigation into the unusual linkage isomerization and nitrite reduction activity of a novel tris(2-pyridyl) copper complex
Source: R Soc Open Sci. 2017 Aug 16;4(8):170593. doi: 10.1098/rsos.170593 (PMC5579116; doi:10.1098/rsos.170593)
Supplement: Symes_Supplementary information_ESM.doc [file rsos170593supp1.docx]

**Supporting Information for:**

An Investigation into the Unusual Linkage Isomerisation and Nitrite Reduction Activity of a Novel Tris(2-pyridyl) Copper Complex

Isolda Roger, Claire Wilson, Hans M. Senn, Stephen Sproules and Mark D. Symes*

WestCHEM, School of Chemistry, University of Glasgow, University Avenue, Glasgow, G12 8QQ, UK.

*E-mail: mark.symes@glasgow.ac.uk

| *Index* | *Page* |
| --- | --- |
| ^1^H and ^13^C NMR spectra of compound **1** | S3 |
| Supplementary calculated infrared spectra | S5 |
| Supplementary EPR Figure S5 | S6 |
| Additional single crystal crystallographic data for [Cu**1**(NO_2_)_2_] (CCDC 1547352)  spectrum of complex **2** in acetonitrile/THF solution at 150 K | S7 |
| References | S15 |
|  |  |

**Figure S1.** ^1^H NMR spectrum of compound **1** in CDCl_3_.

**Figure S2.** Expansion of the aromatic region of the ^1^H NMR spectrum of **1** shown in Fig. S1.

**Figure S3.** ^13^C NMR spectrum of compound **1** in CDCl_3_.

**Supplementary calculated infrared spectra**





**Figure S4.** Calculated infrared spectra of [Cu**1**(NO_2_)_2_]. The cyan curve shows the stretches for the (κ^2^-ONO)(κ^1^-NO_2_) isomer, and the darker blue curve shows the stretches for the (κ^2^-ONO)(κ^1^-ONO) isomer. The red trace shows the sum of the calculated spectra and is also shown in Figure 7 in the main text.


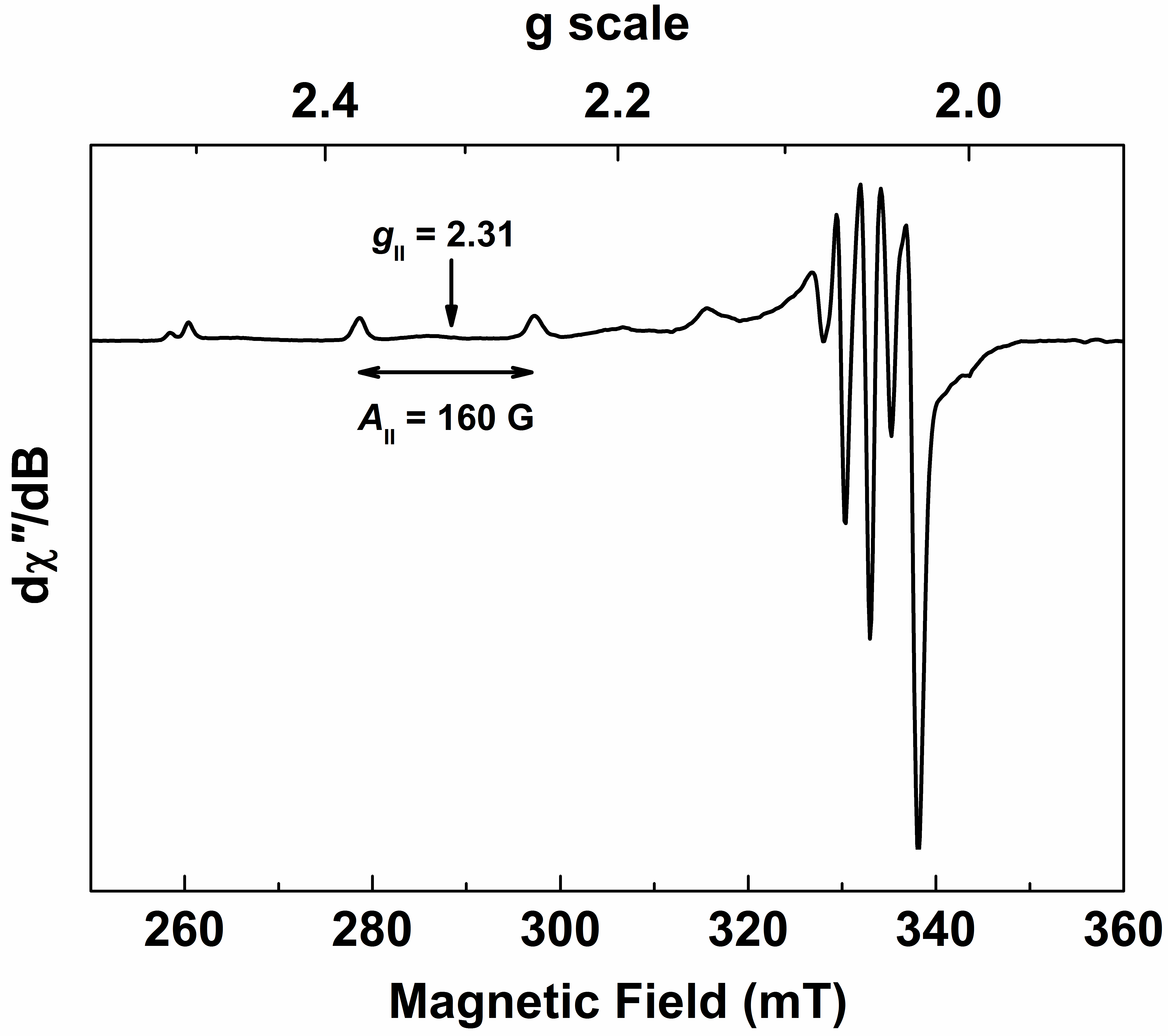


**Figure S5.** X-band EPR spectrum of [Cu**1**(NO_2_)_2_] recorded in MeCN/CH_2_Cl_2_ solution at 130 K (experimental conditions: frequency, 9.4201 GHz; power, 2.0 mW; modulation, 0.07 mT).

**Additional single crystal crystallographic data for [Cu1(NO_2_)_2_] (CCDC 1547352).**

**Refinement**

Crystal data, data collection and structure refinement details are summarised in Table S1.

**Computing details**

Cell refinement: *SAINT* v8.34A (Bruker, 2013); data reduction: *SAINT* v8.34A (Bruker, 2013); program(s) used to refine structure: *SHELXL* (Sheldrick, 2008); molecular graphics: Olex2 (Dolomanov *et al.*, 2009); software used to prepare material for publication: Olex2 (Dolomanov *et al.*, 2009).

**References**

^S^^[[1]](#endnote-2)^Dolomanov, O. V., Bourhis, L. J., Gildea, R. J., Howard, J. A. K. & Puschmann, H. (2009). *J. Appl. Cryst.* **42**, 339–341.

^S^^[[2]](#endnote-3)^Sheldrick, G. M. (2008). *Acta Cryst.* A**64**, 112–122.

*Special details*

| *Refinement*. There is disorder present in both coordinated nitro anions. For one nitro group this is two orientations of the same binding mode present (N1a/b with occupancies approx. 0.55/0.45) and for the other there are two linkage isomers present N2a O-bound and N2b N bound with approx. 0.62/0.38 occupancy. All N—O bonds were restrained to be the same and rigid group displacement ellipsoid restraints were also applied to these atoms. |
| --- |

**(2015gu0007_0m)**

*Crystal data*

| C_18_H_17_CuN_5_O_5_ | *Z* = 2 |
| --- | --- |
| *M_r_* = 446.90 | *F*(000) = 458 |
| Triclinic, *P*¯1 | *D*_x_ = 1.622 Mg m^-3^ |
| *a* = 8.7123 (17) Å | Mo *K*a radiation, l = 0.71073 Å |
| *b* = 8.7388 (17) Å | Cell parameters from 8828 reflections |
| *c* = 14.342 (3) Å | q = 2.7–27.6° |
| a = 72.586 (4)° | m = 1.24 mm^-1^ |
| b = 73.955 (4)° | *T* = 100 K |
| g = 62.928 (4)° | Column, green |
| *V* = 915.0 (3) Å^3^ | 0.46 × 0.1 × 0.06 mm |

*Data collection*

| Bruker APEX-II CCD  diffractometer | 2653 reflections with *I* > 2s(*I*) |
| --- | --- |
| f and w scans | *R*_int_ = 0.079 |
| Absorption correction: multi-scan  *SADABS2012*/1 (Bruker,2012) was used for absorption correction. wR2(int) was 0.1439 before and 0.1107 after correction. The Ratio of minimum to maximum transmission is 0.8308. The l/2 correction factor is 0.0015. | q_max_ = 25.0°, q_min_ = 1.5° |
| *T*_min_ = 0.585, *T*_max_ = 0.704 | *h* = -10®10 |
| 15440 measured reflections | *k* = -10®10 |
| 3197 independent reflections | *l* = -13®17 |

*Refinement*

| Refinement on *F*^2^ | 340 restraints |
| --- | --- |
| Least-squares matrix: full | Hydrogen site location: inferred from neighbouring sites |
| *R*[*F*^2^ > 2s(*F*^2^)] = 0.055 | H-atom parameters constrained |
| *wR*(*F*^2^) = 0.141 | *w* = 1/[s^2^(*F*_o_^2^) + (0.0557*P*)^2^ + 1.9438*P*]  where *P* = (*F*_o_^2^ + 2*F*_c_^2^)/3 |
| *S* = 1.13 | (D/s)_max_ < 0.001 |
| 3197 reflections | Dρ_max_ = 0.74 e Å^-3^ |
| 311 parameters | Dρ_min_ = -0.44 e Å^-3^ |

*Fractional atomic coordinates and isotropic or equivalent isotropic displacement parameters (Å^2^)*

|  | *x* | *y* | *z* | *U*_iso_*/*U*_eq_ | Occ. (<1) |
| --- | --- | --- | --- | --- | --- |
| Cu1 | 0.74845 (7) | 0.73593 (7) | 0.71794 (4) | 0.0252 (2) |  |
| O1 | 1.1176 (4) | 0.8747 (4) | 0.8032 (2) | 0.0233 (7) |  |
| O1A | 0.7409 (15) | 0.6796 (19) | 0.5536 (12) | 0.063 (3) | 0.55 (3) |
| O2A | 0.5356 (19) | 0.823 (2) | 0.6562 (8) | 0.049 (3) | 0.55 (3) |
| O3A | 0.6738 (7) | 0.5462 (7) | 0.7674 (4) | 0.0362 (17) | 0.680 (10) |
| O4A | 0.8201 (5) | 0.4175 (5) | 0.8773 (3) | 0.0422 (9) |  |
| O1B | 0.7318 (15) | 0.718 (2) | 0.5849 (12) | 0.045 (3) | 0.45 (3) |
| O2B | 0.469 (2) | 0.8749 (18) | 0.6297 (11) | 0.053 (4) | 0.45 (3) |
| O3B | 0.6725 (13) | 0.4177 (14) | 0.7742 (8) | 0.036 (3) | 0.320 (10) |
| N1 | 0.7231 (4) | 0.8014 (4) | 0.8541 (2) | 0.0204 (7) |  |
| N2 | 0.7983 (4) | 0.9508 (5) | 0.6562 (2) | 0.0195 (7) |  |
| N3 | 1.0295 (5) | 0.5931 (4) | 0.7083 (2) | 0.0210 (7) |  |
| N1A | 0.580 (2) | 0.764 (6) | 0.578 (2) | 0.058 (8) | 0.55 (3) |
| N2A | 0.7204 (9) | 0.4200 (9) | 0.8357 (6) | 0.0392 (19) | 0.680 (10) |
| N1B | 0.579 (2) | 0.793 (7) | 0.565 (3) | 0.051 (6) | 0.45 (3) |
| N2B | 0.7457 (18) | 0.488 (2) | 0.8009 (9) | 0.024 (3) | 0.320 (10) |
| C1 | 1.0038 (5) | 0.8267 (5) | 0.7755 (3) | 0.0186 (8) |  |
| C2 | 1.0333 (6) | 1.0305 (6) | 0.8447 (3) | 0.0307 (11) |  |
| H2A | 1.0982 | 1.1049 | 0.8150 | 0.046* |  |
| H2B | 1.0309 | 0.9971 | 0.9165 | 0.046* |  |
| H2C | 0.9134 | 1.0953 | 0.8308 | 0.046* |  |
| C11 | 0.8734 (5) | 0.7919 (5) | 0.8678 (3) | 0.0188 (8) |  |
| C12 | 0.9187 (6) | 0.7419 (5) | 0.9606 (3) | 0.0211 (9) |  |
| H12 | 1.0300 | 0.7267 | 0.9681 | 0.025* |  |
| C13 | 0.7982 (6) | 0.7143 (6) | 1.0426 (3) | 0.0251 (9) |  |
| H13 | 0.8265 | 0.6795 | 1.1073 | 0.030* |  |
| C14 | 0.6380 (6) | 0.7373 (6) | 1.0300 (3) | 0.0272 (10) |  |
| H14 | 0.5521 | 0.7250 | 1.0858 | 0.033* |  |
| C15 | 0.6032 (6) | 0.7788 (6) | 0.9343 (3) | 0.0253 (9) |  |
| C16 | 0.4337 (6) | 0.7980 (8) | 0.9174 (4) | 0.0420 (13) |  |
| H16A | 0.4242 | 0.8455 | 0.8471 | 0.063* |  |
| H16B | 0.3373 | 0.8781 | 0.9572 | 0.063* |  |
| H16C | 0.4279 | 0.6830 | 0.9368 | 0.063* |  |
| C21 | 0.9220 (5) | 0.9656 (5) | 0.6873 (3) | 0.0192 (8) |  |
| C22 | 0.9857 (6) | 1.0911 (6) | 0.6363 (3) | 0.0269 (10) |  |
| H22 | 1.0803 | 1.0932 | 0.6558 | 0.032* |  |
| C23 | 0.9122 (6) | 1.2142 (6) | 0.5567 (3) | 0.0333 (11) |  |
| H23 | 0.9530 | 1.3034 | 0.5221 | 0.040* |  |
| C24 | 0.7778 (6) | 1.2042 (6) | 0.5285 (3) | 0.0306 (10) |  |
| H24 | 0.7206 | 1.2898 | 0.4761 | 0.037* |  |
| C25 | 0.7286 (6) | 1.0681 (6) | 0.5776 (3) | 0.0272 (10) |  |
| H25 | 0.6422 | 1.0564 | 0.5553 | 0.033* |  |
| C31 | 1.1177 (5) | 0.6558 (5) | 0.7387 (3) | 0.0184 (8) |  |
| C32 | 1.2967 (6) | 0.5722 (6) | 0.7360 (3) | 0.0248 (9) |  |
| H32 | 1.3563 | 0.6189 | 0.7584 | 0.030* |  |
| C33 | 1.3871 (6) | 0.4185 (6) | 0.6997 (3) | 0.0327 (11) |  |
| H33 | 1.5101 | 0.3577 | 0.6970 | 0.039* |  |
| C34 | 1.2952 (7) | 0.3549 (6) | 0.6676 (3) | 0.0344 (11) |  |
| H34 | 1.3543 | 0.2503 | 0.6420 | 0.041* |  |
| C35 | 1.1186 (6) | 0.4450 (6) | 0.6731 (3) | 0.0295 (10) |  |
| H35 | 1.0563 | 0.4008 | 0.6511 | 0.035* |  |

*Atomic displacement parameters (Å^2^)*

|  | *U*^11^ | *U*^22^ | *U*^33^ | *U*^12^ | *U*^13^ | *U*^23^ |
| --- | --- | --- | --- | --- | --- | --- |
| Cu1 | 0.0252 (3) | 0.0384 (4) | 0.0193 (3) | -0.0182 (3) | -0.0024 (2) | -0.0083 (2) |
| O1 | 0.0237 (15) | 0.0315 (17) | 0.0211 (15) | -0.0158 (13) | -0.0036 (12) | -0.0068 (13) |
| O1A | 0.078 (6) | 0.102 (7) | 0.043 (7) | -0.066 (5) | 0.000 (4) | -0.024 (5) |
| O2A | 0.044 (5) | 0.088 (7) | 0.027 (4) | -0.040 (5) | -0.014 (3) | -0.003 (4) |
| O3A | 0.035 (3) | 0.039 (3) | 0.040 (3) | -0.020 (3) | -0.009 (2) | -0.005 (2) |
| O4A | 0.050 (2) | 0.039 (2) | 0.040 (2) | -0.0204 (18) | -0.0141 (16) | -0.0016 (16) |
| O1B | 0.051 (5) | 0.075 (7) | 0.030 (5) | -0.045 (5) | -0.008 (4) | -0.007 (4) |
| O2B | 0.046 (7) | 0.076 (7) | 0.034 (6) | -0.036 (6) | -0.020 (5) | 0.022 (5) |
| O3B | 0.035 (6) | 0.037 (7) | 0.047 (7) | -0.019 (5) | -0.020 (5) | -0.005 (5) |
| N1 | 0.0192 (17) | 0.0249 (19) | 0.0157 (16) | -0.0102 (15) | -0.0021 (13) | -0.0006 (14) |
| N2 | 0.0139 (16) | 0.0279 (19) | 0.0139 (16) | -0.0056 (14) | 0.0000 (13) | -0.0070 (14) |
| N3 | 0.0278 (18) | 0.0220 (18) | 0.0130 (16) | -0.0104 (15) | -0.0014 (14) | -0.0045 (14) |
| N1A | 0.077 (7) | 0.108 (17) | 0.023 (7) | -0.069 (6) | -0.008 (4) | -0.008 (11) |
| N2A | 0.028 (4) | 0.039 (4) | 0.053 (4) | -0.010 (3) | -0.006 (3) | -0.018 (3) |
| N1B | 0.053 (7) | 0.087 (12) | 0.036 (9) | -0.054 (6) | -0.019 (4) | 0.010 (9) |
| N2B | 0.016 (6) | 0.029 (6) | 0.028 (5) | -0.010 (5) | -0.003 (4) | -0.007 (4) |
| C1 | 0.0177 (19) | 0.025 (2) | 0.0145 (18) | -0.0096 (17) | -0.0048 (15) | -0.0033 (15) |
| C2 | 0.038 (3) | 0.035 (3) | 0.029 (2) | -0.023 (2) | -0.002 (2) | -0.011 (2) |
| C11 | 0.022 (2) | 0.019 (2) | 0.0154 (18) | -0.0070 (17) | -0.0016 (15) | -0.0063 (15) |
| C12 | 0.024 (2) | 0.022 (2) | 0.0186 (19) | -0.0103 (18) | -0.0051 (16) | -0.0036 (16) |
| C13 | 0.032 (2) | 0.026 (2) | 0.015 (2) | -0.0108 (19) | -0.0036 (17) | -0.0039 (17) |
| C14 | 0.028 (2) | 0.031 (3) | 0.020 (2) | -0.013 (2) | 0.0031 (17) | -0.0060 (18) |
| C15 | 0.022 (2) | 0.030 (2) | 0.021 (2) | -0.0090 (19) | 0.0007 (16) | -0.0064 (17) |
| C16 | 0.028 (3) | 0.062 (4) | 0.035 (3) | -0.019 (2) | 0.001 (2) | -0.012 (3) |
| C21 | 0.018 (2) | 0.023 (2) | 0.0117 (18) | -0.0051 (17) | 0.0019 (15) | -0.0068 (15) |
| C22 | 0.031 (2) | 0.031 (2) | 0.020 (2) | -0.016 (2) | 0.0007 (18) | -0.0064 (17) |
| C23 | 0.043 (3) | 0.034 (3) | 0.018 (2) | -0.017 (2) | -0.0003 (19) | -0.0005 (19) |
| C24 | 0.029 (2) | 0.034 (3) | 0.015 (2) | -0.005 (2) | -0.0003 (18) | -0.0033 (18) |
| C25 | 0.019 (2) | 0.037 (3) | 0.016 (2) | -0.0033 (19) | -0.0033 (16) | -0.0056 (17) |
| C31 | 0.0217 (19) | 0.022 (2) | 0.0084 (18) | -0.0094 (16) | -0.0003 (15) | 0.0008 (15) |
| C32 | 0.024 (2) | 0.030 (2) | 0.016 (2) | -0.0109 (18) | -0.0001 (16) | -0.0017 (17) |
| C33 | 0.027 (2) | 0.036 (3) | 0.020 (2) | -0.005 (2) | 0.0021 (18) | -0.0030 (19) |
| C34 | 0.042 (3) | 0.029 (3) | 0.022 (2) | -0.006 (2) | 0.0012 (19) | -0.0087 (19) |
| C35 | 0.043 (3) | 0.028 (2) | 0.018 (2) | -0.015 (2) | -0.0048 (18) | -0.0055 (18) |

*Geometric parameters (Å, º) for (2015gu0007_0m)*

| Cu1—O2A | 2.005 (16) | C2—H2C | 0.9800 |
| --- | --- | --- | --- |
| Cu1—O3A | 1.922 (5) | C11—C12 | 1.379 (6) |
| Cu1—O1B | 2.010 (18) | C12—H12 | 0.9500 |
| Cu1—N1 | 2.122 (3) | C12—C13 | 1.386 (6) |
| Cu1—N2 | 2.009 (3) | C13—H13 | 0.9500 |
| Cu1—N3 | 2.169 (4) | C13—C14 | 1.372 (6) |
| Cu1—N2B | 2.147 (14) | C14—H14 | 0.9500 |
| O1—C1 | 1.419 (5) | C14—C15 | 1.393 (6) |
| O1—C2 | 1.443 (5) | C15—C16 | 1.489 (6) |
| O1A—N1A | 1.255 (12) | C16—H16A | 0.9800 |
| O2A—N1A | 1.264 (11) | C16—H16B | 0.9800 |
| O3A—N2A | 1.227 (8) | C16—H16C | 0.9800 |
| O4A—N2A | 1.171 (7) | C21—C22 | 1.377 (6) |
| O4A—N2B | 1.276 (12) | C22—H22 | 0.9500 |
| O1B—N1B | 1.258 (13) | C22—C23 | 1.383 (6) |
| O2B—N1B | 1.251 (13) | C23—H23 | 0.9500 |
| O3B—N2B | 1.249 (12) | C23—C24 | 1.385 (7) |
| N1—C11 | 1.339 (5) | C24—H24 | 0.9500 |
| N1—C15 | 1.357 (5) | C24—C25 | 1.375 (7) |
| N2—C21 | 1.344 (5) | C25—H25 | 0.9500 |
| N2—C25 | 1.345 (5) | C31—C32 | 1.383 (6) |
| N3—C31 | 1.339 (5) | C32—H32 | 0.9500 |
| N3—C35 | 1.338 (6) | C32—C33 | 1.387 (7) |
| C1—C11 | 1.542 (5) | C33—H33 | 0.9500 |
| C1—C21 | 1.540 (5) | C33—C34 | 1.388 (7) |
| C1—C31 | 1.525 (6) | C34—H34 | 0.9500 |
| C2—H2A | 0.9800 | C34—C35 | 1.364 (7) |
| C2—H2B | 0.9800 | C35—H35 | 0.9500 |
|  |  |  |  |
| O2A—Cu1—N1 | 119.5 (3) | C12—C11—C1 | 119.6 (4) |
| O2A—Cu1—N2 | 95.3 (4) | C11—C12—H12 | 120.7 |
| O2A—Cu1—N3 | 148.2 (3) | C11—C12—C13 | 118.6 (4) |
| O3A—Cu1—O2A | 76.8 (4) | C13—C12—H12 | 120.7 |
| O3A—Cu1—N1 | 99.38 (19) | C12—C13—H13 | 120.1 |
| O3A—Cu1—N2 | 172.10 (19) | C14—C13—C12 | 119.7 (4) |
| O3A—Cu1—N3 | 100.9 (2) | C14—C13—H13 | 120.1 |
| O1B—Cu1—N1 | 168.9 (4) | C13—C14—H14 | 120.5 |
| O1B—Cu1—N3 | 97.3 (4) | C13—C14—C15 | 119.1 (4) |
| O1B—Cu1—N2B | 98.4 (5) | C15—C14—H14 | 120.5 |
| N1—Cu1—N3 | 92.30 (13) | N1—C15—C14 | 121.0 (4) |
| N1—Cu1—N2B | 88.1 (3) | N1—C15—C16 | 118.3 (4) |
| N2—Cu1—O1B | 90.7 (4) | C14—C15—C16 | 120.7 (4) |
| N2—Cu1—N1 | 84.50 (13) | C15—C16—H16A | 109.5 |
| N2—Cu1—N3 | 85.73 (13) | C15—C16—H16B | 109.5 |
| N2—Cu1—N2B | 166.6 (3) | C15—C16—H16C | 109.5 |
| N2B—Cu1—N3 | 83.4 (4) | H16A—C16—H16B | 109.5 |
| C1—O1—C2 | 115.2 (3) | H16A—C16—H16C | 109.5 |
| N1A—O2A—Cu1 | 108.6 (10) | H16B—C16—H16C | 109.5 |
| N2A—O3A—Cu1 | 128.3 (5) | N2—C21—C1 | 118.1 (3) |
| N1B—O1B—Cu1 | 114.1 (14) | N2—C21—C22 | 120.9 (4) |
| C11—N1—Cu1 | 112.3 (3) | C22—C21—C1 | 120.7 (4) |
| C11—N1—C15 | 119.1 (3) | C21—C22—H22 | 119.9 |
| C15—N1—Cu1 | 122.8 (3) | C21—C22—C23 | 120.2 (4) |
| C21—N2—Cu1 | 118.0 (3) | C23—C22—H22 | 119.9 |
| C21—N2—C25 | 119.0 (4) | C22—C23—H23 | 120.8 |
| C25—N2—Cu1 | 122.5 (3) | C22—C23—C24 | 118.4 (4) |
| C31—N3—Cu1 | 118.6 (3) | C24—C23—H23 | 120.8 |
| C35—N3—Cu1 | 122.8 (3) | C23—C24—H24 | 120.6 |
| C35—N3—C31 | 118.5 (4) | C25—C24—C23 | 118.8 (4) |
| O1A—N1A—O2A | 115.8 (15) | C25—C24—H24 | 120.6 |
| O4A—N2A—O3A | 115.6 (7) | N2—C25—C24 | 122.4 (4) |
| O2B—N1B—O1B | 113.1 (16) | N2—C25—H25 | 118.8 |
| O4A—N2B—Cu1 | 117.4 (7) | C24—C25—H25 | 118.8 |
| O3B—N2B—Cu1 | 120.2 (10) | N3—C31—C1 | 114.1 (3) |
| O3B—N2B—O4A | 122.4 (13) | N3—C31—C32 | 122.4 (4) |
| O1—C1—C11 | 109.1 (3) | C32—C31—C1 | 123.5 (4) |
| O1—C1—C21 | 109.4 (3) | C31—C32—H32 | 120.8 |
| O1—C1—C31 | 106.5 (3) | C31—C32—C33 | 118.4 (4) |
| C21—C1—C11 | 115.7 (3) | C33—C32—H32 | 120.8 |
| C31—C1—C11 | 108.7 (3) | C32—C33—H33 | 120.5 |
| C31—C1—C21 | 107.1 (3) | C32—C33—C34 | 119.0 (4) |
| O1—C2—H2A | 109.5 | C34—C33—H33 | 120.5 |
| O1—C2—H2B | 109.5 | C33—C34—H34 | 120.5 |
| O1—C2—H2C | 109.5 | C35—C34—C33 | 118.9 (4) |
| H2A—C2—H2B | 109.5 | C35—C34—H34 | 120.5 |
| H2A—C2—H2C | 109.5 | N3—C35—C34 | 122.8 (4) |
| H2B—C2—H2C | 109.5 | N3—C35—H35 | 118.6 |
| N1—C11—C1 | 118.1 (3) | C34—C35—H35 | 118.6 |
| N1—C11—C12 | 122.2 (4) |  |  |
|  |  |  |  |
| Cu1—O2A—N1A—O1A | -5 (5) | C11—C1—C21—N2 | -50.5 (5) |
| Cu1—O3A—N2A—O4A | 1.9 (10) | C11—C1—C21—C22 | 135.5 (4) |
| Cu1—O1B—N1B—O2B | 5 (6) | C11—C1—C31—N3 | 63.4 (4) |
| Cu1—N1—C11—C1 | 29.0 (4) | C11—C1—C31—C32 | -117.0 (4) |
| Cu1—N1—C11—C12 | -146.9 (3) | C11—C12—C13—C14 | -0.1 (6) |
| Cu1—N1—C15—C14 | 147.6 (4) | C12—C13—C14—C15 | 3.5 (7) |
| Cu1—N1—C15—C16 | -31.8 (5) | C13—C14—C15—N1 | -1.8 (7) |
| Cu1—N2—C21—C1 | -6.2 (5) | C13—C14—C15—C16 | 177.6 (4) |
| Cu1—N2—C21—C22 | 167.8 (3) | C15—N1—C11—C1 | -177.1 (4) |
| Cu1—N2—C25—C24 | -172.5 (3) | C15—N1—C11—C12 | 6.9 (6) |
| Cu1—N3—C31—C1 | -1.0 (4) | C21—N2—C25—C24 | -0.8 (6) |
| Cu1—N3—C31—C32 | 179.4 (3) | C21—C1—C11—N1 | 34.7 (5) |
| Cu1—N3—C35—C34 | -179.7 (3) | C21—C1—C11—C12 | -149.2 (4) |
| O1—C1—C11—N1 | 158.5 (3) | C21—C1—C31—N3 | -62.3 (4) |
| O1—C1—C11—C12 | -25.4 (5) | C21—C1—C31—C32 | 117.3 (4) |
| O1—C1—C21—N2 | -174.2 (3) | C21—C22—C23—C24 | -1.8 (7) |
| O1—C1—C21—C22 | 11.8 (5) | C22—C23—C24—C25 | -3.0 (7) |
| O1—C1—C31—N3 | -179.3 (3) | C23—C24—C25—N2 | 4.4 (7) |
| O1—C1—C31—C32 | 0.4 (5) | C25—N2—C21—C1 | -178.2 (3) |
| N1—C11—C12—C13 | -5.2 (6) | C25—N2—C21—C22 | -4.3 (6) |
| N2—C21—C22—C23 | 5.6 (6) | C31—N3—C35—C34 | 0.4 (6) |
| N3—C31—C32—C33 | 0.4 (6) | C31—C1—C11—N1 | -85.8 (4) |
| C1—C11—C12—C13 | 178.9 (4) | C31—C1—C11—C12 | 90.3 (4) |
| C1—C21—C22—C23 | 179.4 (4) | C31—C1—C21—N2 | 70.8 (4) |
| C1—C31—C32—C33 | -179.2 (4) | C31—C1—C21—C22 | -103.2 (4) |
| C2—O1—C1—C11 | -60.2 (4) | C31—C32—C33—C34 | 0.1 (6) |
| C2—O1—C1—C21 | 67.3 (4) | C32—C33—C34—C35 | -0.4 (6) |
| C2—O1—C1—C31 | -177.3 (3) | C33—C34—C35—N3 | 0.1 (7) |
| C11—N1—C15—C14 | -3.3 (6) | C35—N3—C31—C1 | 178.9 (3) |
| C11—N1—C15—C16 | 177.3 (4) | C35—N3—C31—C32 | -0.7 (6) |

Document origin: *publCIF*.^S^^[[3]](#endnote-4)^

1. S O. V. Dolomanov, L. J. Bourhis, R. J. Gildea, J. A. K. Howard and H. Puschmann, *J. Appl. Cryst.* 2009, **42**, 339. [↑](#endnote-ref-2)
2. S G. M. Sheldrick, *Acta Cryst.* 2008, A**64**, 112–122 [↑](#endnote-ref-3)
3. S S. P. Westrip, *J. Apply. Cryst.*, 2010, **43**, 920. [↑](#endnote-ref-4)
